# Cholangioscopy-guided tunneling and coaxial stenting of a large choledocholithiasis: a novel approach to mechanical lithotripsy

**DOI:** 10.1016/j.vgie.2023.09.002

**Published:** 2023-09-09

**Authors:** Abdulrahman Qatomah, Abrar Nawawi, Abdullah Albishi, Yeganeh Rostamian Moghaddam, Corey Miller, Yen-I Chen

**Affiliations:** 1 Division of Gastroenterology and Hepatology, McGill University Health Centre, Montreal, Quebec, Canada; 2 Division of Gastroenterology and Hepatology, Jewish General Hospital, McGill University, Montreal, Quebec, Canada; 3 Division of Gastroenterology and Hepatology, McGill University Health Centre, Montreal, Quebec, Canada

## Abstract

Video 1 Cholangioscopy-guided tunneling and coaxial stenting of a large choledocholithiasis: a novel approach to mechanical lithotripsy. Fluoroscopy image of initial ERCP shows large filling defect (*blue circle*) and guidewire passing toward the common hepatic duct (*black arrow*).

Cholangioscopy-guided tunneling and coaxial stenting of a large choledocholithiasis: a novel approach to mechanical lithotripsy. Fluoroscopy image of initial ERCP shows large filling defect (*blue circle*) and guidewire passing toward the common hepatic duct (*black arrow*).

Single-operator cholangioscopy-guided electrohydraulic lithotripsy (SOC-EHL) has greatly improved the rate of ductal clearance of large challenging stones with ERCP.^1^, ^2^, ^3^ Nevertheless, this approach sometimes fails to remove the stones. The following describes a novel method to ductal clearance for a large choledocholithiasis refractory to conventional ERCP and SOC-EHL.

A 62-year-old woman presented to the emergency department with fever and jaundice. Her initial labs showed a bilirubin level of 116 μ/L and white blood cell count of 15.8 10 × 9/L. A CT scan of the abdomen revealed a 2.3-cm calcified common bile duct stone (Fig. 1). Initial ERCP failed ductal clearance with sphincterotomy and dilation-assisted stone extraction using a 13-mm balloon for a duration of 30 seconds. SOC-EHL was then performed but failed to accomplish significant stone fragmentation with 2 probes (Fig. 2).

**Figure 1:**
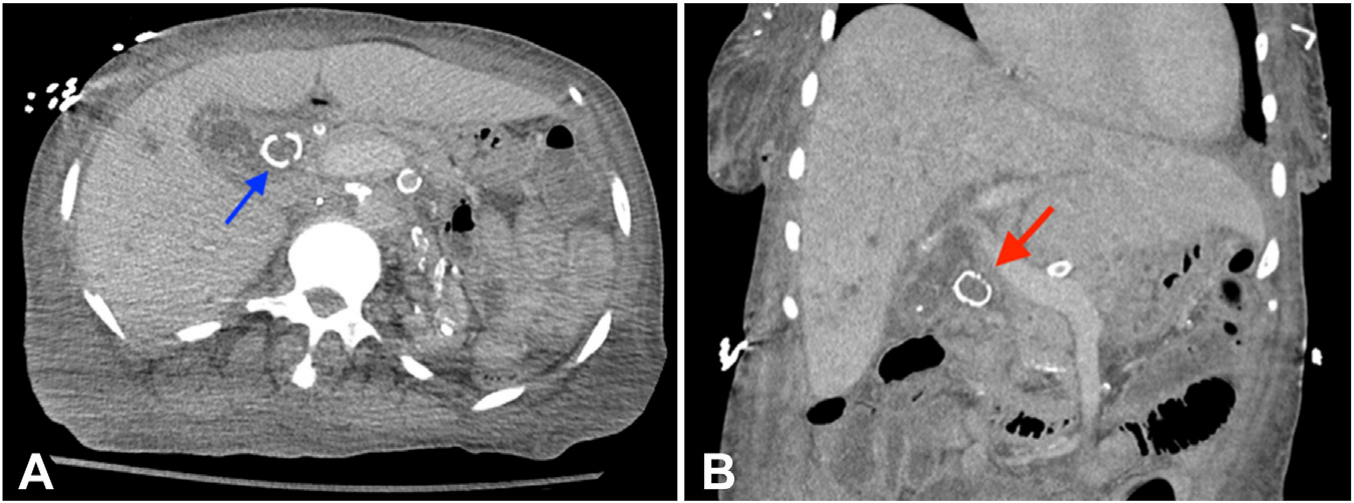
**A,** Abdominal CT scan (*axial*) showing a large, calcified common bile duct (CBD) stone (*blue arrow*). **B,** Abdominal CT scan (*coronal*) showing a large calcified CBD stone (*red arrow*).

**Figure 2:**
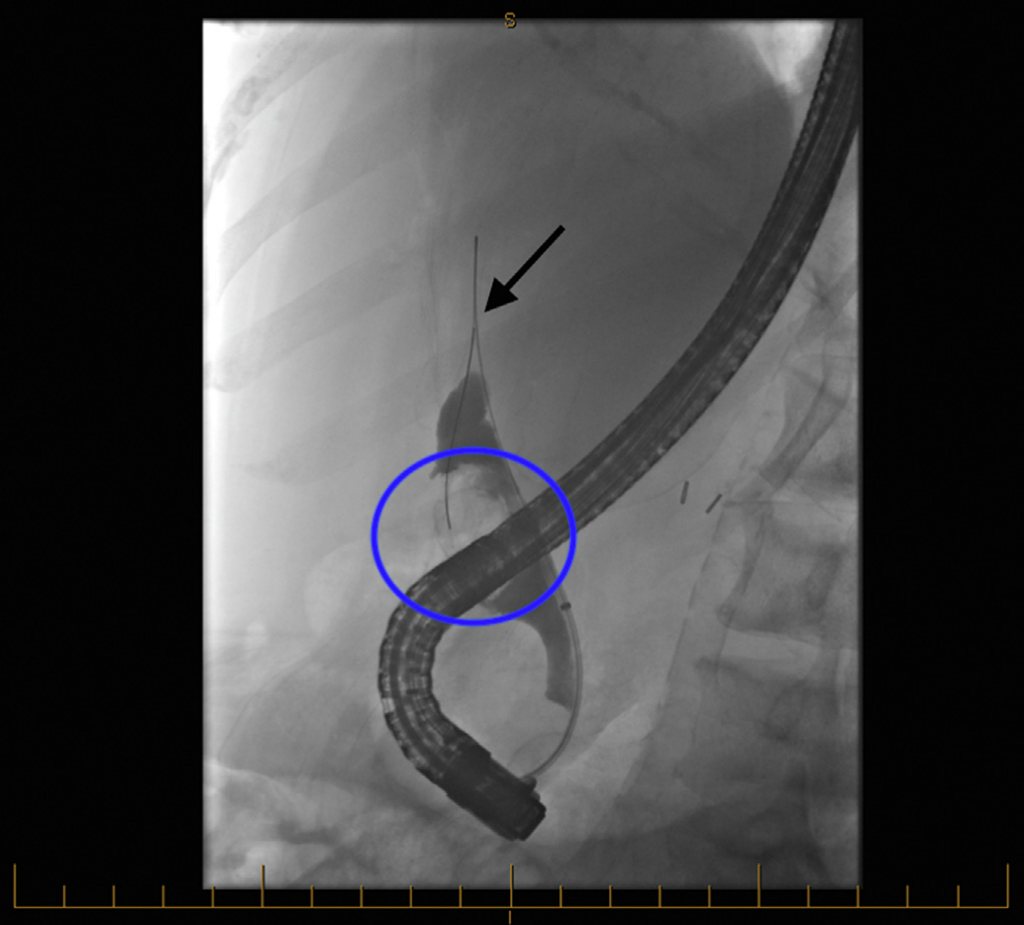
Fluoroscopy image of initial ERCP shows large filling defect (*blue circle*) and guidewire passing toward the common hepatic duct (*black arrow*).

Using SOC-EHL with a total of 4 EHL probes, we created a small tunnel across the large stone. Under SOC guidance, a 0.035-inch guidewire was advanced through this tunnel into the intrahepatic biliary tree. A fully covered self-expandable metal stent, 10 mm × 80F, was then deployed through the stone for mechanical lithotripsy (Fig. 3). ERCP was then repeated 3 weeks later. The metal stent was removed and had weakened the core of the stone, which allowed for ease of complete fragmentation of the calcified choledocholithiasis using SOC-EHL. Complete ductal clearance was achieved with balloon sweeps (Video 1, available online at www.videogie.org).

**Figure 3:**
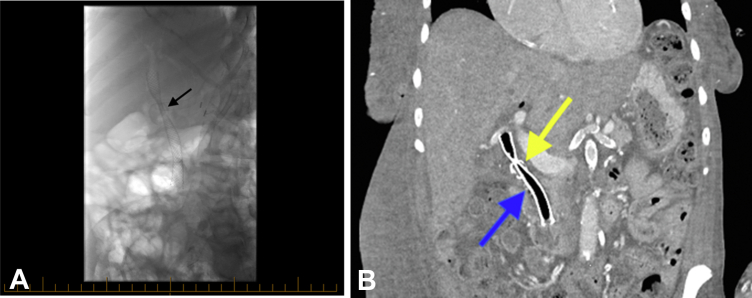
**A,** Fluoroscopy image following insertion of a self-expandable metal stent (SEMS) through the common bile duct (CBD) stone (*black arrow*). **B,** Abdominal CT scan (*coronal*) showing SEMS (*blue arrow*) passing through the CBD stone (*yellow arrow*).

In cases of large choledocholithiasis refractory to standard ERCP and SOC-EHL, SOC-EHL–guided stone tunneling followed by coaxial metal stent insertion exerts a radial force (mechanical lithotripsy) on the stone to weaken its core, allowing subsequent stone fragmentation and ductal clearance (Fig. 4).

**Figure 4:**
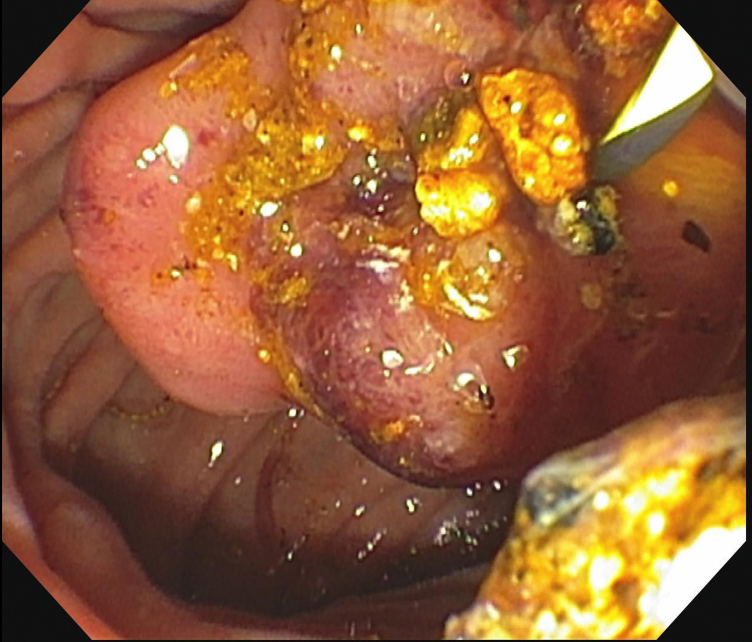
Endoscopic image of a fragmented stone after laser lithotripsy.

## Disclosure

Dr Chen is a consultant for Boston Scientific and is the president of Chess Medical, Inc. The other authors did not disclose any financial relationships.

## Supplementary data

Video 1 Cholangioscopy-guided tunneling and coaxial stenting of a large choledocholithiasis: a novel approach to mechanical lithotripsy. Fluoroscopy image of initial ERCP shows large filling defect (*blue circle*) and guidewire passing toward the common hepatic duct (*black arrow*).
